# The Practice Market.—VI
*The previous articles in this series were published in The Hospital of March 18, March 25, April 1, April 29,
and June 17.


**Published:** 1911-06-24

**Authors:** 


### SPECIAL ARTICLE.

### THE PRACTICE MARKET.—VI.*

### FURTHER CONSIDERATIONS FOR THE PURCHASER.

In our last article we urged the prospective buyer in the practice market to think out exactly what he wants and how much he can pay for it before he applies to an agent or takes any other definite step in the way of finding an opening. It has been pointed out to us that many junior practitioners who are anxious to settle down are in need of advice before they can make up their minds to the best advantage about the kind of work that will suit them. This week, therefore, we will consider some of the pros and cons of partnership and practices, and of general practice in town and country. After that we will discuss the procedure with agencies, the limitations of the medical agent, and the investigation of practices.

Practice *v.* Partnership.

In dealing with the rival claims of partnership and practice, it must first be said that, weight for weight, a good partnership involves a larger outlay than a good practice, because the risk is proportionately less. A practice gives the newcomer independence; he is his own master, so far as that can be said of any medical man. He will have no disagreements with his partners or their wives, and his earnings will be his own. But he may need a *locum tenens* for every holiday, even a week-end, and for every day that he is ill—and *locums* are expensive and not always reliable. When in difficulties he will depend for assistance upon a professional opponent, while if he is single-handed in a rural district help in time of trouble may be far away.

A partnership in an established firm offers various advantages to the junior man. He learns the complicated ropes of general practice under the guidance of an experienced colleague, who will initiate him into methods of business and the general conduct of the practice. He can obtain help in emergencies, advice in tactical difficulties, and assistance in operations and midwifery work, while anæsthetics and consultations are always available. Moreover, illness and holidays are robbed of half their perils, since one partner is usually able to relieve another, at least for a time. On the other hand, a partnership may be one long series of irritations, jealousies and misunderstandings. Medical firms of three, four, and even five partners are more common nowadays than they used to be, especially around

* The previous articles in this series were published in The Hospital of March 18, March 25, April 1, April 29, and June 17.

London, and sometimes they are extremely successful in every respect. Some authorities recommend such large joint practices as being the best of all openings, but the junior partners may have to remain many years in a subordinate position and without appreciable rise of income. Three partners are often said to work better together than two, the third acting as a buffer in disagreements.

### Country Practice.

As to choice of locality, a rural or country town practice is naturally best suited to a man with open-air tastes. In rural practice there is often little opposition, and there is usually a good prospect of the transference of "goodwill" and appointments, while the newcomer soon becomes known socially and professionally to the neighbourhood. Against these advantages must be set off the long distances and exposure to all weathers, the absence of help and the preponderance of poor-class work, which are part of village doctoring. Country-town practice combines the pros and cons of town and country; there is more security and fresh air than in city work, and less stagnation than in rural practice. Cottage hospitals, with well-equipped operating theatres and trained nursing, have added greatly to the scope and attractiveness of country-town work for able and ambitious practitioners. Country practice, with few exceptions, is essentially "mixed," and dispensing is at present almost inevitable where parish, club, and dispensary work is undertaken.

### Suburban and Town Practices.

Suburban practices correspond most closely to those in the larger country towns, but competition is perhaps keener and the standard of professional skill is perhaps rather higher. On the other hand, responsibility may be a little less, because expert help and advice are more accessible and less costly. The suburbs are not so isolated from the heart of things, but some fastidious men find the suburban atmosphere unendurable, while country life and society are much more to their taste. South-country men with London diplomas should think deeply before settling upon openings in northern cities or their environs, where local University degrees are often at a premium.

Apart from "lock-up" or "cash" practices it may be said that most good-class practices in the heart of London require private means of the purchaser. Family practice seems to be dying out in the Metropolis, where patients are prone to fly to another doctor, or a dozen doctors, without reason or notice, and visit specialists on the least provocation. Introductions, therefore, however promising and *bona fide*, may lead to little transfer of work, and a good practice may melt away in a few months after the vendor has gone, in spite of his best offices. London practices are intensely personal, and partnerships are not common in the West End. Patients are liable to send for an opponent if their own attendant is away or ill, and partnership introductions are therefore of less value; the incoming partner must try to work up his own connection. For these reasons many advise the junior man bent on London practice not to sink money in a vacancy or a share, but to put up his plate and wait. But there is heavy competition in all London districts, and neighbouring practitioners do not view a "squatter" with cordiality. London practice, except of the "sixpenny" sort, is essentially for the man with a private income and a circle of London acquaintances.

